# Primary Small Cell Carcinoma of the Vagina

**DOI:** 10.1155/2013/827037

**Published:** 2013-06-25

**Authors:** Rafael Oliveira, Mayra Coelho Bócoli, João Carlos Saldanha, Eddie Fernando Candido Murta, Rosekeila Simões Nomelini

**Affiliations:** ^1^Research Institute of Oncology (IPON), Discipline of Gynecology and Obstetrics, Federal University of Triângulo Mineiro (UFTM), Avenida Getúlio Guaritá 214, 38025-440 Uberaba, MG, Brazil; ^2^Discipline of Surgical Pathology, Federal University of Triângulo Mineiro (UFTM), Uberaba, MG, Brazil

## Abstract

The primary small cell carcinoma of the vagina is rare, and it is a highly aggressive malignancy with no consensus regarding the treatment of this tumor. The survival rate for patients treated in the early stages is around two years. We related the case report of a patient of 41 years with a vegetative and necrotic lesion in left vaginal wall, in middle and upper third, and involvement of parametrium in its proximal third and medium third. A biopsy showed a small cell undifferentiated carcinoma composed of epithelial cells with round nuclei, oval or elongated, hyperchromatic nuclei, with little distinct nucleoli, and scarce cytoplasm. Immunohistochemistry showed positivity for AE1/AE3, CD57, and chromogranin A. The patient received 6 cycles of chemotherapy with cisplatin and etoposide and radiotherapy, achieving complete response, with complete regression of the lesion. The patient had no sign of tumor recurrence and locoregional or distant metastases after 5 months of followup.

## 1. Introduction 

Primary malignant epithelial tumors of the vagina can be divided into three types: adenocarcinoma, squamous cell carcinoma, and small cell carcinoma. The primary small cell carcinoma of the vagina is rare, and it is a highly aggressive malignancy [1]. The survival rate for patients treated in the early stages is around two years [2]. There is no consensus regarding the treatment of this tumor. Furthermore, the treatment follows the model treatment of small cell carcinoma of the lung, which is the most common primary site. The therapies most commonly performed are radiotherapy in combination with chemotherapy and, in some cases, radical hysterectomy and vaginectomy, with the appearance of distant metastases several months after the treatment [3]. The most common symptoms presented by patients are vaginal discharge and pelvic pain [4]. 

## 2. Case Presentation

**Figure 1:**
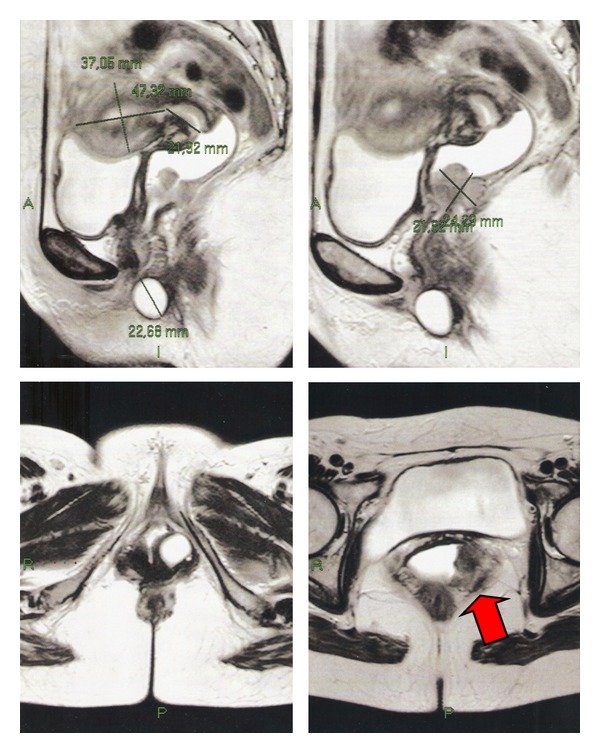
Magnetic resonance imaging: a solid mass in the middle third of the posterior wall of the vagina with invasion of left parametrium.

**Figure 2:**
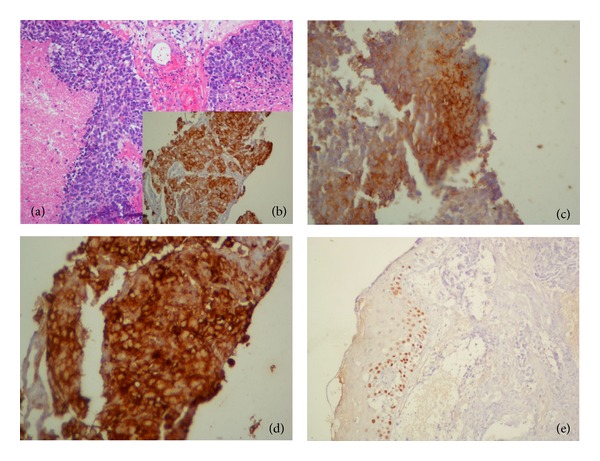
Hematoxylin-eosin stain (a), AE1/AE3 (b), CD57 (c), chromogranin A (d), and p63 (e).

A patient of 41 years sought gynecology service in May 2012 complaining of vaginal discharge, yellow foul-smelling vaginal discharge for about 1 month. The speculum examination revealed a vegetative and necrotic lesion with papillary appearance in left vaginal wall, in middle and upper third. Rectal examination showed involvement of parametrium in its proximal third and medium third. The colposcopic examination showed a lesion with atypical vascularization, white epithelium, and Lugol-negative area. Magnetic resonance imaging demonstrated a solid mass, heterogeneous and vegetating in the middle third of the posterior wall of the vagina measuring 2.4 × 2.1 cm with invasion of left parametrium (Figure 1). A biopsy of the left vaginal wall showed a small cell undifferentiated carcinoma. The malignant neoplasm is composed of epithelial cells with round nuclei, oval or elongated, hyperchromatic nuclei, with little distinct nucleoli, and scarce cytoplasm. There were numerous atypical mitoses, extensive areas of necrosis and karyorrhexis. Immunohistochemistry showed positivity for AE1/AE3, CD57, and chromogranin A (Figure 2), and its expression was negative for CD56, p63, and NSE (neuron-specific enolase), confirming the diagnosis of small cell carcinoma of the vagina. The X-ray and computed tomography of the chest and upper abdomen showed no lesions suggestive of metastases. The patient received 6 cycles of chemotherapy with cisplatin and etoposide and radiotherapy (teletherapy and brachytherapy), achieving complete response, with complete regression of the lesion. The patient had no sign of tumor recurrence and locoregional or distant metastases after 5 months of followup.

## 3. Discussion

The small cell carcinoma accounts for 2% of malignancies diagnosed in the female genital tract. Its most common primary site is the lung, which represents 95% of all diagnosed cases [3]. The most common site of involvement of the female genital tract is the cervix, followed by the ovary, endometrium, vagina, and, finally, the vulva [5]. The primary site in our patient was the vagina, since imaging showed no primary disease in most common sites such as the lungs.

Albores Saavedra et al. documented the first small cell neuroendocrine carcinoma of the lower female genital tract in 1972, as a carcinoid tumor of the uterine cervix [6]. The first small cell carcinoma with vaginal primary site was diagnosed in 1984 by Scully et al. [7]. This primary site is rare for small cell carcinoma, with 28 cases reported in the literature [3]. The primary small cell carcinoma of the vagina manifests with vaginal bleeding or metastatic symptoms [8], or by leucorrhoea, as in our patient. Our patient is younger than the average age of 56 found in the review and clinicopathological study of Bing et al. [9] of 23 patients, but was within the range of 38 to 74 years. In this review, four patients were in stage I, eight in stage II, five in stage III, and four in stage IV. There was no information about the staging of the two patients. Our patient was in stage II. According to Bing et al. [9], 5 of 18 patients presented with information regarding lymph node metastasis or distant at diagnosis. Our patient had no evidence of distant metastasis at diagnosis.

The rarity of small cell carcinoma is responsible for the uncertainty of the best therapy and it is based on the treatment for small cell carcinoma of the lung. Cases of small cell carcinoma restricted to the lung have better responses with chemoradiotherapy compared to chemotherapy alone, both in complete remission and in survival [10, 11]. When chemoradiotherapy is started early, the local response and disease-free survival are better [12, 13]. Thoracic radiotherapy performed twice daily with chemotherapy with cisplatin and etoposide was associated with increased survival. However, esophagitis grade 3 becomes more frequent [14]. The acute toxicity in the perineum limits the radiotherapy twice daily with chemotherapy [5]. 

Local therapy alone in small cell carcinoma of the vagina is associated with poor survival, requiring a combination of therapies, as with small cell carcinoma in other primary sites. However, there is a greater frequency of side effects in the pelvic region [8]. Initial treatment is chemotherapy associated with radiotherapy or surgical resection associated with adjuvant chemotherapy and radiotherapy. Small lesion in the upper third of the vagina can be treated by radical hysterectomy, partial vaginectomy, and pelvic lymphadenectomy [5, 9]. 

The treatment regimens for small cell carcinoma of the vagina used in the cases reported by the review of Bing et al. [9] were radiochemotherapy (11 patients), radiotherapy alone (4 patients), chemotherapy alone (3 patients), surgery alone (2 patients), and association of chemoradiotherapy and surgery (1 patient). There was no description of treating in two patients. The combination of chemotherapy and radiotherapy was the treatment of choice with greater evidence of complete cure after treatment completion. The main chemotherapy drugs used were cisplatin and etoposide [3]. Our patient underwent chemotherapy with cisplatin and etoposide and subsequent radiotherapy. 

The initial response of small cell carcinoma radiotherapy and/or chemotherapy is excellent; however, distant recurrence is common [15], being the main cause of mortality [5]. Gardner et al. [16] reported that 85% of patients with small cell carcinoma of the vagina die within a year of diagnosis. Twenty-three patients were analyzed for review by Bing et al. [9], fifteen patients died in an average of twelve months after the end of treatment (range, from five to 29 months), four were alive and only one of them had signs of injury until the publication of the case report (still in treatment), and two were only described as having the pathology of small cell carcinoma. The four living patients were in stage I. Of the 20 patients analyzed for review by Kaminski et al. [5], fifteen patients died at a mean of five months after the end of treatment (range, from 5 to 12 months), three were alive and without signs of injury until the publication of the case report, and two were only described as having the pathology of small cell carcinoma. The three patients who had not progressed to death were in stage I.

Small cell carcinomas are clinically aggressive, with rapid recurrences and distant metastases. The correct diagnosis is important in establishing treatment.
